# Ethyl 5-formyl-3,4-dimethyl-1*H*-pyrrole-2-carboxyl­ate

**DOI:** 10.1107/S160053680902323X

**Published:** 2009-06-20

**Authors:** Wei-Na Wu, Yuan Wang, Qiu-Fen Wang

**Affiliations:** a Department of Physics and Chemistry, Henan Polytechnic University, Jiaozuo 454000, People’s Republic of China

## Abstract

The mol­ecule of the title compound, C_10_H_13_NO_3_, is approximately planar (maximum deviation 0.1424 Å). In the crystal, mol­ecules are linked into inversion dimers by pairs of N—H⋯O hydrogen bonds, and the dimeric units are linked by non-classical C—H⋯O hydrogen bonds, forming a layered structure.

## Related literature

For a related structure, see: Kang *et al.* (2008 ▶). For our studies of bis­(pyrrol-2-yl-methyl­eneamine) ligands, see: Wang *et al.* (2008 ▶, 2009 ▶). For the synthesis, see: Wang *et al.* (2008 ▶). 
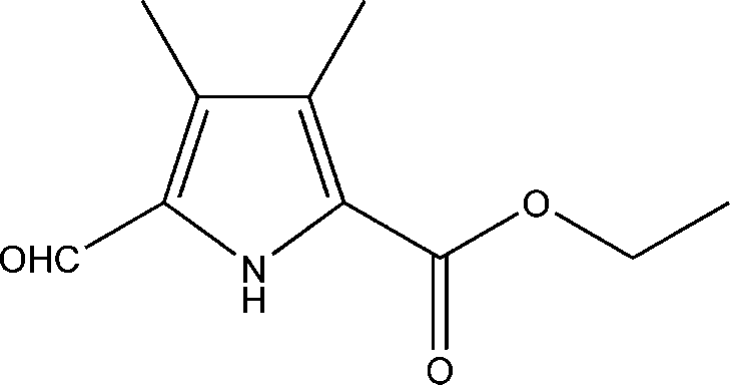


## Experimental

### 

#### Crystal data

C_10_H_13_NO_3_
*M* *_r_* = 195.21
Triclinic, 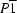

*a* = 7.2223 (12) Å
*b* = 7.4347 (12) Å
*c* = 10.0488 (17) Å
α = 78.412 (2)°
β = 84.191 (2)°
γ = 79.051 (2)°
*V* = 517.84 (15) Å^3^
*Z* = 2
Mo *K*α radiation
μ = 0.09 mm^−1^
*T* = 296 K
0.30 × 0.18 × 0.15 mm

#### Data collection

Bruker SMART CCD diffractometer
Absorption correction: multi-scan (*SADABS*; Sheldrick, 1996 ▶) *T*_min_ = 0.980, *T*_max_ = 0.986
6232 measured reflections
2416 independent reflections
1692 reflections with *I* > 2σ(*I*)
*R*_int_ = 0.017

#### Refinement

*R*[*F* ^2^ > 2σ(*F* ^2^)] = 0.060
*wR*(*F* ^2^) = 0.212
*S* = 1.07
2416 reflections
129 parameters
H-atom parameters constrained
Δρ_max_ = 0.29 e Å^−3^
Δρ_min_ = −0.29 e Å^−3^

### 

Data collection: *SMART* (Bruker, 2001 ▶); cell refinement: *SAINT* (Bruker, 2001 ▶); data reduction: *SAINT*; program(s) used to solve structure: *SHELXL97* (Sheldrick, 2008 ▶); program(s) used to refine structure: *SHELXL97* (Sheldrick, 2008 ▶); molecular graphics: *SHELXTL* (Sheldrick, 2008 ▶); software used to prepare material for publication: *SHELXTL*.

## Supplementary Material

Crystal structure: contains datablocks I, global. DOI: 10.1107/S160053680902323X/gw2066sup1.cif

Structure factors: contains datablocks I. DOI: 10.1107/S160053680902323X/gw2066Isup2.hkl

Additional supplementary materials:  crystallographic information; 3D view; checkCIF report

## Figures and Tables

**Table 1:** Hydrogen-bond geometry (Å, °)

*D*—H⋯*A*	*D*—H	H⋯*A*	*D*⋯*A*	*D*—H⋯*A*
N1—H1*A*⋯O2^i^	0.86	2.07	2.919 (2)	169
C5—H5*A*⋯O1^ii^	0.93	2.54	3.347 (3)	145

Symmetry codes: (i) 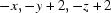; (ii) 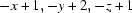.

**Fig. 1.**
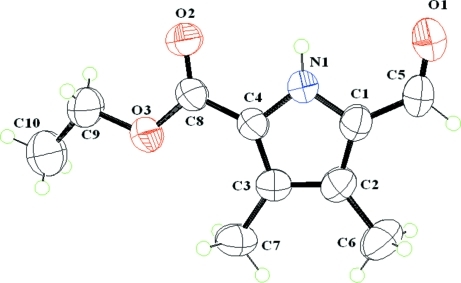
The molecular structure of the title compound shown with 50% probability displacement ellipsoids.

**Fig. 2.**
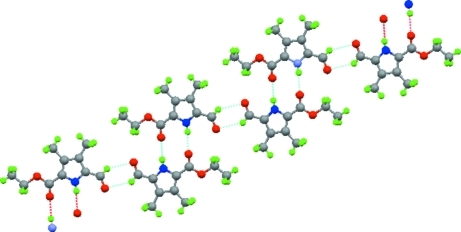
Layered structure of the title compound. Hydrogen-bonding interactions are shown as dashed lines.

## supplementary crystallographic information

### Comment

Schiff Base bearing pyrrole units have been extensively investigated for a long time because they could stabilize mono- or binuclear metal complexes which have various structures and special properties. As part of our owning studies of bis(pyrrol-2-yl-methyleneamine) ligands (Wang *et al.*, 2008), we report here the crystal structure of the title compound, (I), (Fig. 1), which is approximately planar.

The molecules are joined to dimers *via* intermolecular N—H···O hydrogen bonds (Table 1), and the dimeric units are linked with each other by nonclassical C—H···O hydrogen bonds (Table 1) to form a layered geometry (Fig. 2).

### Experimental

A quantity of POCl_3_ (2.30 g, 0.015 mol) was added dropwise to DMF (1.10 g, 0.015 mol) under stirring on an ice-water bath, then a CH_2_Cl_2 _solution (30 ml) containing 3,4-dimethyl-2-ethoxycarbonyl-pyrrole (2.51 g, 0.015 mol) was added. After stirring at room temperature for 4 h, a 10% Na_2_CO_3_ solution (80 ml) was added. The reaction mixture was refluxing for 0.5 h, then cooled to room temperature, extracted with CH_2_Cl_2 _ (3×10 ml), and dried with anhydrous Na_2_CO_3_. The solvent was evaporated under reduced pressure. The crude product was treated with column chromatography on silica gel [petroleum ether-ethyl acetate (100:1)] to yield (I) 2.25 g (77%). Colorless prisms of (I) were obtained by slow evaporation of an ethanol solution.

### Refinement

(type here to add refinement details)

### Crystal data

**Table:**

C_10_H_13_NO_3_	*Z* = 2
*M**_r_* = 195.21	*F*(000) = 208
Triclinic, *P*1	*D*_x_ = 1.252 Mg m^−^^3^
*a* = 7.2223 (12) Å	Mo *K*α radiation, λ = 0.71073 Å
*b* = 7.4347 (12) Å	Cell parameters from 2123 reflections
*c* = 10.0488 (17) Å	θ = 2.1–27.7°
α = 78.412 (2)°	µ = 0.09 mm^−^^1^
β = 84.191 (2)°	*T* = 296 K
γ = 79.051 (2)°	Prism, colorless
*V* = 517.84 (15) Å^3^	0.30 × 0.18 × 0.15 mm

### Data collection

**Table:**

Bruker SMART CCD diffractometer	2416 independent reflections
Radiation source: fine-focus sealed tube	1692 reflections with *I* > 2σ(*I*)
graphite	*R*_int_ = 0.017
φ and ω scans	θ_max_ = 27.7°, θ_min_ = 2.1°
Absorption correction: multi-scan (*SADABS*; Sheldrick, 1996)	*h* = −9→9
*T*_min_ = 0.980, *T*_max_ = 0.986	*k* = −9→9
6232 measured reflections	*l* = −13→13

### Refinement

**Table:**

Refinement on *F*^2^	Primary atom site location: structure-invariant direct methods
Least-squares matrix: full	Secondary atom site location: difference Fourier map
*R*[*F*^2^ > 2σ(*F*^2^)] = 0.060	Hydrogen site location: inferred from neighbouring sites
*wR*(*F*^2^) = 0.212	H-atom parameters constrained
*S* = 1.07	*w* = 1/[σ^2^(*F*_o_^2^) + (0.1226*P*)^2^ + 0.0669*P*] where *P* = (*F*_o_^2^ + 2*F*_c_^2^)/3
2416 reflections	(Δ/σ)_max_ < 0.001
129 parameters	Δρ_max_ = 0.29 e Å^−^^3^
0 restraints	Δρ_min_ = −0.29 e Å^−^^3^

### Special details

**Table:**

Geometry. All e.s.d.'s (except the e.s.d. in the dihedral angle between two l.s. planes) are estimated using the full covariance matrix. The cell e.s.d.'s are taken into account individually in the estimation of e.s.d.'s in distances, angles and torsion angles; correlations between e.s.d.'s in cell parameters are only used when they are defined by crystal symmetry. An approximate (isotropic) treatment of cell e.s.d.'s is used for estimating e.s.d.'s involving l.s. planes.
Refinement. Refinement of *F*^2^ against ALL reflections. The weighted *R*-factor *wR* and goodness of fit *S* are based on *F*^2^, conventional *R*-factors *R* are based on *F*, with *F* set to zero for negative *F*^2^. The threshold expression of *F*^2^ > σ(*F*^2^) is used only for calculating *R*-factors(gt) *etc*. and is not relevant to the choice of reflections for refinement. *R*-factors based on *F*^2^ are statistically about twice as large as those based on *F*, and *R*- factors based on ALL data will be even larger.

### Fractional atomic coordinates and isotropic or equivalent isotropic displacement parameters (Å^2^)

**Table:**

	*x*	*y*	*z*	*U*_iso_*/*U*_eq_	
C4	0.3634 (3)	0.8003 (2)	1.03572 (18)	0.0519 (5)	
C8	0.2197 (3)	0.8074 (3)	1.1488 (2)	0.0593 (5)	
C3	0.5533 (3)	0.7196 (2)	1.0262 (2)	0.0556 (5)	
C1	0.4613 (3)	0.8683 (3)	0.8185 (2)	0.0585 (5)	
C2	0.6149 (3)	0.7620 (3)	0.8883 (2)	0.0605 (5)	
C5	0.4495 (4)	0.9519 (4)	0.6766 (2)	0.0780 (7)	
H5A	0.5582	0.9328	0.6195	0.094*	
C7	0.6715 (3)	0.6084 (3)	1.1392 (2)	0.0720 (6)	
H7A	0.7978	0.5703	1.1030	0.108*	
H7B	0.6181	0.5001	1.1808	0.108*	
H7C	0.6741	0.6836	1.2060	0.108*	
C9	0.1496 (4)	0.7387 (5)	1.3863 (3)	0.1023 (10)	
H9A	0.1126	0.8660	1.4008	0.123*	
H9B	0.0375	0.6948	1.3693	0.123*	
C10	0.2342 (5)	0.6221 (6)	1.5051 (3)	0.1191 (12)	
H10A	0.1453	0.6265	1.5826	0.179*	
H10B	0.3450	0.6663	1.5212	0.179*	
H10C	0.2687	0.4960	1.4906	0.179*	
C6	0.8106 (3)	0.7039 (4)	0.8285 (3)	0.0872 (8)	
H6A	0.8884	0.6318	0.8993	0.131*	
H6B	0.8631	0.8128	0.7869	0.131*	
H6C	0.8059	0.6301	0.7612	0.131*	
O3	0.2874 (2)	0.7315 (2)	1.26943 (15)	0.0771 (5)	
O2	0.0572 (2)	0.8726 (3)	1.13418 (17)	0.0899 (6)	
O1	0.3095 (3)	1.0448 (3)	0.62612 (17)	0.1053 (7)	
N1	0.3105 (2)	0.8889 (2)	0.90931 (15)	0.0539 (4)	
H1A	0.1993	0.9484	0.8903	0.065*	

### Atomic displacement parameters (Å^2^)

**Table:**

	*U*^11^	*U*^22^	*U*^33^	*U*^12^	*U*^13^	*U*^23^
C4	0.0506 (9)	0.0554 (9)	0.0481 (10)	−0.0087 (7)	−0.0052 (7)	−0.0051 (7)
C8	0.0550 (11)	0.0682 (11)	0.0493 (10)	−0.0082 (8)	−0.0053 (8)	0.0004 (8)
C3	0.0517 (10)	0.0526 (9)	0.0619 (11)	−0.0072 (7)	−0.0068 (8)	−0.0095 (8)
C1	0.0601 (11)	0.0638 (11)	0.0518 (11)	−0.0101 (8)	0.0033 (8)	−0.0155 (8)
C2	0.0555 (11)	0.0608 (10)	0.0652 (12)	−0.0072 (8)	0.0024 (9)	−0.0180 (9)
C5	0.0779 (15)	0.1027 (17)	0.0473 (12)	−0.0053 (13)	0.0084 (11)	−0.0156 (11)
C7	0.0584 (12)	0.0679 (12)	0.0837 (15)	0.0005 (9)	−0.0165 (10)	−0.0048 (10)
C9	0.0778 (16)	0.148 (3)	0.0571 (14)	−0.0005 (16)	0.0093 (12)	0.0119 (14)
C10	0.108 (2)	0.161 (3)	0.0668 (17)	−0.010 (2)	0.0026 (16)	0.0126 (18)
C6	0.0651 (14)	0.0912 (16)	0.0983 (19)	−0.0010 (12)	0.0157 (13)	−0.0233 (14)
O3	0.0625 (9)	0.1094 (12)	0.0475 (8)	−0.0070 (8)	−0.0043 (7)	0.0061 (7)
O2	0.0552 (9)	0.1288 (15)	0.0616 (10)	0.0092 (9)	0.0013 (7)	0.0126 (9)
O1	0.0927 (14)	0.1528 (19)	0.0505 (10)	0.0128 (12)	−0.0006 (9)	−0.0065 (10)
N1	0.0507 (8)	0.0637 (9)	0.0445 (8)	−0.0055 (7)	−0.0028 (6)	−0.0083 (6)

### Geometric parameters (Å, °)

**Table:**

C4—N1	1.364 (2)	C7—H7B	0.9600
C4—C3	1.390 (3)	C7—H7C	0.9600
C4—C8	1.461 (3)	C9—C10	1.443 (4)
C8—O2	1.194 (2)	C9—O3	1.463 (3)
C8—O3	1.330 (2)	C9—H9A	0.9700
C3—C2	1.405 (3)	C9—H9B	0.9700
C3—C7	1.500 (3)	C10—H10A	0.9600
C1—N1	1.354 (2)	C10—H10B	0.9600
C1—C2	1.396 (3)	C10—H10C	0.9600
C1—C5	1.440 (3)	C6—H6A	0.9600
C2—C6	1.498 (3)	C6—H6B	0.9600
C5—O1	1.206 (3)	C6—H6C	0.9600
C5—H5A	0.9300	N1—H1A	0.8600
C7—H7A	0.9600		
			
N1—C4—C3	108.93 (17)	H7B—C7—H7C	109.5
N1—C4—C8	117.47 (17)	C10—C9—O3	108.7 (3)
C3—C4—C8	133.61 (18)	C10—C9—H9A	109.9
O2—C8—O3	123.29 (19)	O3—C9—H9A	109.9
O2—C8—C4	123.38 (19)	C10—C9—H9B	109.9
O3—C8—C4	113.32 (17)	O3—C9—H9B	109.9
C4—C3—C2	106.33 (17)	H9A—C9—H9B	108.3
C4—C3—C7	127.64 (19)	C9—C10—H10A	109.5
C2—C3—C7	126.03 (19)	C9—C10—H10B	109.5
N1—C1—C2	108.25 (17)	H10A—C10—H10B	109.5
N1—C1—C5	121.70 (19)	C9—C10—H10C	109.5
C2—C1—C5	130.0 (2)	H10A—C10—H10C	109.5
C1—C2—C3	107.43 (18)	H10B—C10—H10C	109.5
C1—C2—C6	126.8 (2)	C2—C6—H6A	109.5
C3—C2—C6	125.8 (2)	C2—C6—H6B	109.5
O1—C5—C1	125.0 (2)	H6A—C6—H6B	109.5
O1—C5—H5A	117.5	C2—C6—H6C	109.5
C1—C5—H5A	117.5	H6A—C6—H6C	109.5
C3—C7—H7A	109.5	H6B—C6—H6C	109.5
C3—C7—H7B	109.5	C8—O3—C9	115.39 (18)
H7A—C7—H7B	109.5	C1—N1—C4	109.06 (16)
C3—C7—H7C	109.5	C1—N1—H1A	125.5
H7A—C7—H7C	109.5	C4—N1—H1A	125.5
			
N1—C4—C8—O2	6.4 (3)	C7—C3—C2—C1	−179.66 (18)
C3—C4—C8—O2	−173.7 (2)	C4—C3—C2—C6	−179.9 (2)
N1—C4—C8—O3	−174.52 (16)	C7—C3—C2—C6	−0.1 (3)
C3—C4—C8—O3	5.4 (3)	N1—C1—C5—O1	−0.2 (4)
N1—C4—C3—C2	−0.1 (2)	C2—C1—C5—O1	−178.7 (2)
C8—C4—C3—C2	179.9 (2)	O2—C8—O3—C9	−2.1 (4)
N1—C4—C3—C7	−179.99 (18)	C4—C8—O3—C9	178.8 (2)
C8—C4—C3—C7	0.1 (3)	C10—C9—O3—C8	169.4 (3)
N1—C1—C2—C3	−0.7 (2)	C2—C1—N1—C4	0.6 (2)
C5—C1—C2—C3	178.0 (2)	C5—C1—N1—C4	−178.23 (18)
N1—C1—C2—C6	179.8 (2)	C3—C4—N1—C1	−0.3 (2)
C5—C1—C2—C6	−1.5 (4)	C8—C4—N1—C1	179.68 (15)
C4—C3—C2—C1	0.5 (2)		

### Hydrogen-bond geometry (Å, °)

**Table:**

*D*—H···*A*	*D*—H	H···*A*	*D*···*A*	*D*—H···*A*
N1—H1A···O2^i^	0.86	2.07	2.919 (2)	169
C5—H5A···O1^ii^	0.93	2.54	3.347 (3)	145

Symmetry codes: (i) −*x*, −*y*+2, −*z*+2; (ii) −*x*+1, −*y*+2, −*z*+1.
